# The occurrence of elephant endotheliotropic herpesvirus infection in wild and captive Asian elephants in Thailand: Investigation based on viral DNA and host antibody

**DOI:** 10.14202/vetworld.2021.545-550

**Published:** 2021-02-27

**Authors:** Phirom Prompiram, Witthawat Wiriyarat, Benjaporn Bhusri, Weena Paungpin, Waleemas Jairak, Supaphen Sripiboon, Tuempong Wongtawan

**Affiliations:** 1 The Monitoring and Surveillance Centre for Zoonotic Diseases in Wildlife and Exotic Animals, Faculty of Veterinary Science, Mahidol University, Salaya, Phutthamonthon, Nakhon Pathom, 73170, Thailand; 2 Laboratory of Veterinary Biomedicine, Faculty of Veterinary Science, Mahidol University, Salaya, Phutthamonthon, Nakhon Pathom, 73170, Thailand; 3 Department of Preclinical Science, Mahidol University, Salaya, Phutthamonthon, Nakhon Pathom, 73170, Thailand; 4 Zoological Park Organization, 267 Pracharat 1 Road, Bangsue Bangkok 10800, Thailand; 5 Department of Large Animal and Wildlife Clinical Sciences, Faculty of Veterinary Medicine, Kasetsart University, Kamphaeng Saen Campus, Nakhon Pathom 73140, Thailand; 6 Centre of Excellence for One Health, Akkhraratchakumari Veterinary College, Walailak University, Thai Buri, Tha Sala, Nakhon Si Thammarat 80160 Thailand; 7 Centre of Excellence Research for Melioidosis and other Microorganism, Walailak University, Thai Buri, Tha Sala, Nakhon Si Thammarat 80160 Thailand

**Keywords:** Carrier, elephant endotheliotropic herpesvirus, elephant, enzyme-linked immunosorbent assay, polymerase chain reaction

## Abstract

**Background and Aim:**

Elephant endotheliotropic herpesvirus (EEHV) is a serious disease, threatening the life of young elephants. Many elephants have been infected with no clinical signs and may serve as carriers spreading this disease. It is important to monitor the disease through clinical signs and molecular diagnosis. In this study we investigated the occurrence of EEHV and the efficiency of different techniques used to monitor EEHV infection in various samples and populations of Asian elephants.

**Materials and Methods:**

Blood and trunk swabs were collected from live elephants, while visceral organs (lung, digestive tract, spleen, lymph nodes, and kidney) were collected from dead elephants. EEHV was detected by polymerase chain reaction (PCR) in whole blood, trunk swabs, and visceral organs as samples, while elephant anti-EEHV immunoglobulin G (IgG) in serum was detected by enzyme-linked immunosorbent assay (ELISA). A total of 162 samples were analyzed in this study: 129 from healthy, 26 from dead, and 7 from sick elephants.

**Results:**

The present study showed that the overall incidence of EEHV was 40.1% (n=65/162). Approximately 46.2% (n=12/26) and 85.7% (n=6/7) of dead and sick elephants were positive for EEHV by PCR, respectively. All sick elephants that were young and affected by EEHV clinical disease tested negative for the IgG antibody ELISA, suggesting primary EEHV infection in this group. In addition, 2.3% (n=3/129) of subclinical infections were detected using PCR, and trunk swab samples showed slightly higher sensitivity (5.3%, n=2/38) to detect EEHV than whole blood (1.2%, n=1/84). As many as, 48.4% (n=44/91) of healthy elephants were EEHV seropositive (ELISA-positive), suggesting that many elephants in Thailand had previously been infected. Overall, 30% of dead wild elephants had been infected with EEHV (n=3/10). Moreover, statistical analysis revealed no significant differences in the EEHV detection rate between different age groups or sexes (p>0.05).

**Conclusion:**

PCR is better than ELISA to detect EEHV active infection in dead/sick elephants and to monitor EEHV in young elephants. ELISA is suitable for detecting previous EEHV infection and carriers, particularly adults. Theoretically, we could use both PCR and ELISA to increase the sensitivity of testing, along with observing abnormal behavior to efficiently monitor this disease. Identification of EEHV carriers within elephant populations is important to prevent transmission to healthy individuals, especially young elephants with high mortality from EEHV. This is the first report from Thailand regarding EEHV infection in wild elephants, showing the importance of preventing disease transmission between captive and wild elephants.

## Introduction

Elephant endotheliotropic herpesvirus (EEHV) is a serious disease that can threaten the life of young elephants [1,2]. No vaccine is currently available for this disease and its pathogenesis is poorly understood. The symptoms are varied and depend on the elephant’s age and the type of EEHV. EEHV-infected adult elephants (older than 15 years old) mostly show no clinical signs, whereas most young elephants infected by EEHV exhibit serious illness, potentially leading to death [3,4]. Among the seven types of EEHV, type 1 is the most likely to be lethal by causing acute hemorrhagic syndrome. Although most adult elephants do not exhibit symptoms of EEHV, they can be carriers of the disease and transmit the virus to young elephants [3-5]. The common subtypes found in clinical EEHV cases in Thailand are EEHV1A (60%), EEHV4 (29%), and EEHV1B (11%) [3].

The conventional method of diagnosing EEHV is by polymerase chain reaction (PCR) to detect viral DNA. Whole blood is commonly used to identify EEHV by PCR in living animals, while EEHV in dead animals is detected within visceral organs [6-9]. Routine monitoring of EEHV in elephant herds is important to detect preclinical viremia for early medical intervention and to identify carriers to prevent disease transmission. From routine monitoring, the occurrence of EEHV in healthy elephants (carriers) was found to be between 1.78% and 5.5%, as detected by PCR using whole blood samples [6,7]. This suggests that PCR detection alone might not be sufficiently sensitive to detect EEHV infection due toits reliance on the viremia period to allow viruses to move out from host cells, and also require frequent monitoring. Alternatively, PCR can be used to detect EEHV from cells obtained from trunk wash, which appears to provide higher sensitivity than whole blood. Although this method is not convenient for most elephants because it requires elephant training, samples from trunk wash or swabs could indicate shedding of the virus from elephants and potential transmission of the virus through contact with at-risk juveniles [8,10,11]. Another method to detect EEHV infection is through the detection of EEHV antibody (immunoglobulin G [IgG]) by enzyme-linked immunosorbent assay (ELISA), but only one group has recently reported the effectiveness of EEHV detection by ELISA [12]. In that previous study, EEHV incidence, as tested by ELISA, was approximately 60% in European and US zoos [12], and 40% in captive elephants in Thailand [13]. Another method for EEHV detection is *in situ* hybridization; it is not convenient for use as a conventional diagnostic method, although it is more useful for studying the disease mechanisms [14].

In this study, we investigated the occurrence of EEHV and the efficiency of PCR and ELISA techniques to monitor EEHV infection in various samples (whole blood, trunk swab, visceral organ, and serum) and diverse populations of Asian elephants such as both sexes, different age groups, as well as captive and wild elephants.

## Materials and Methods

### Ethical approval

This experiment was approved by the Animal Care and Use Committee of the University (Protocol Number: MUVS-2020-20).

### Study period and location

All samples were collected and analyzed between 2000 and 2020. EEHV diagnosis was performed by Mahidol University, Kasetsart University, and Zoological Park Organization. The study areas were Bangkok, Kanchanaburi, Chonburi, Ayutthaya, Prachuap Khiri Khan, Phetchaburi, Ratchaburi, and Surin provinces.

### Samples

Samples were collected from 162 Asian elephants: 10 samples from wild elephants and 152 from captive ones. Twenty-six samples were collected from elephants suspected to have died from EEHV, 7 samples were collected from sick elephants whose illness was suspected to be due to EEHV, and 129 samples were collected from healthy elephants.

Blood samples (whole blood and serum) from live elephants were obtained from sample banks of the universities and zoos. The whole blood was used to detect viral DNA while serum was used to detect EEHV antibody. Visceral organs (lung, digestive tract, spleen, lymph nodes, and kidney) of EEHV suspected-dead elephants were sent to the Faculty of Veterinary Science to detect EEHV.

### PCR

Viral DNA was extracted from whole blood and tissues using the phenol-chloroform method. The viral DNA was detected by either nested PCR and electrophoresis or quantitative PCR, in accordance with the previous studies [6,11,15], to detect EEHV types 1-7.

### ELISA

ELISA was performed at the Monitoring and Surveillance Centre for Zoonotic Diseases in Wildlife and Exotic Animals (MoZWE). Three peptides were used as antigens. These antigens were designed using BepiPred-2.0, the B-cell epitope prediction program [16], based on the glycoprotein B sequence (GenBank: AAG41998.1). The peptide sequences were GDNDKKFSETYTKFKVYNEYERLE, ANMTKHRRKRETSSSASSK, and QQHVGDPPSYDESIGSSHTYSK. The antigens were coated on a MaxiSorp^®^ 96-well microplate (Thermo Fisher Scientific, Massachusetts, USA), incubated at 4°C overnight, washed 5 times with 0.05% Tween 20 in phosphate-buffered saline (PBS-T) (Thermo Scientific, Massachusetts, USA), and blocked with 5% skim milk in PBS (Fluka; Sigma-Aldrich, Missouri, USA) at 37°C for 1 h. Thereafter, 100 μL of diluted elephant serum (1:100 in blocking solution) was added and incubated at 37°C for 1 h before washing with PBS-T 5 times. Specific elephant antibodies were captured with 100 μL of diluted (1:1000 in blocking solution) rabbit anti-elephant IgG polyclonal antibody (in-house preparation) at 37°C for 1 h and washed with PBS-T. Subsequently, 100 μL of mouse anti-rabbit IgG monoclonal antibody conjugated with horseradish peroxidase (SouthernBiotech, Alabama, USA) in blocking solution (1:500) was added and incubated at 37°C for 1 h, and then washed with PBS-T. Next, 100 μL of KPL-TMB peroxidase solution was added and incubated at room temperature (25°C) for 15 min in a dark box; the reaction was stopped by adding 1 N NaOH. The absorbance was read at 450 nm using an ELx808 ELISA plate reader and KC Junior program (Biotek Instrument, Vermont, USA). The cutoff was the mean of the negative control plus 0.2, and samples were considered positive if their mean was higher than the cutoff. Each reaction was performed in duplicate. The validity of this ELISA was verified using positive and negative samples. Positive samples were serum from elephants with a history of EEHV infection (PCR positive); they recently tested positive by ELISA in a previous study [13]. Negative samples were collected from elephants with no history of EEHV infection, which tested negative by both PCR and ELISA [6,7,13].

### Statistical analysis

Fisher’s exact test with two-tailed analysis was used to calculate p values and identify significant differences between the groups. The web-based statistical software GraphPad (GraphPad Software, California, USA) was used.

## Results

### Occurrence of EEHV detected by PCR and ELISA

The rates of identification of EEHV in this study are shown in Table-1. In total, 162 samples were tested, 40.1% (n=65/162) of which were positive for EEHV. Most of the sick elephants suspected of having EEHV were PCR positive (85.7%; n=6/7), but none of them were ELISA positive (seropositive). All of the sick elephants were younger than 9 years old.

**Table-1:** Occurrence of EEHV detected by PCR and ELISA in EEHV suspected dead or sick elephant and healthy elephants.

EEHV test/sample	Dead (n)	Sick (n)	Healthy (n) with or without EEHV record^#^			Total (n)

			With	Without		
			
	Visceral organs	Blood	Blood	Blood	Trunk swap	
PCR positive	46.2% (12/26)	85.7% (6/7)	0.0% (0/7)	1.2% (1/84)	5.3% (2/38)	13.0% (21/162)
ELISA positive	*	0.0% (0/7)	100% (7/7)	44.0% (37/84)	*	43.9% (43/98)^1^
All positive	46.2% (12/26)	85.7% (6/7)	100% (7/7)	45.2% (38/84)	5.3% (2/38)	40.1% (65/162)
All negative	53.8% (14/26)	14.3% (1/7)	0.0% (0/7)	55.8% (46/84)	94.7% (36/38)	59.9% (97/162)
Total	100% (26/26)	100% (7/7)	100% (7/7)	100% (84/84)	100% (38/38)	100% (162/162)

* ELISA could not be used to detect EEHV from visceral organs and trunk swab.

# EEHV record means elephants may be previously infected with EEHV, confirmed by PCR, or observed clinical sign.

1 Total number of ELISA test was 98(blood from sick elephants and healthy elephants). EEHV=Elephant endotheliotropic herpesvirus, PCR=Polymerase chain reaction, ELISA=Enzyme linked immunosorbent assay

For the 26 dead elephants suspected of having been infected with EEHV, we found that 46.2% (n=12/26) of samples were PCR positive. Ten of the dead elephants were wild elephants, 30% of which were EEHV positive (n=3/10). We found that two of the dead wild elephants had been infected with EEHV1, while another had been infected with EEHV4.

We obtained 129 healthy elephant samples, among which 36.4% were EEHV positive (n=47/129); PCR detected EEHV infection at a rate of 2.3% (n=3/129), while ELISA did so at 48.4% (n=44/91). The median age of elephants showing ELISA positivity was 25 years old, with the youngest being 3 years old. Seven of these samples were collected from elephants that had been infected with EEHV many years previously (from the records), and they all tested positive by ELISA (100%; n=7/7), but not by PCR, in the present study. For the 122 elephants with no record of EEHV infection (both clinical signs and PCR test), our results showed that their blood samples were PCR positive at a rate of 1.2% (n=1/84) and ELISA positive at 43.9% (n=43/98). Their trunk swabs were PCR positive at a rate of 5.3% (n=2/38), whereas the majority of healthy elephants remained negative for EEHV. In all healthy elephants, EEHV infection was detected much more frequently (p<0.001) by ELISA (43.9%; n=43/98) than by PCR (2.3%; n=3/129).

### Comparisons by sex and age for EEHV infection in healthy elephants with no record of EEHV

Because we only had complete data of age and sex in the group of captive healthy elephants that had no record of EEHV, we could only compare the occurrence of EEHV in healthy elephants with regard to age and sex (Table-2). The data suggested that adult elephants tended to be infected by EEHV more than young elephants and male elephants tended to be infected more than females. However, statistical analysis revealed no significant differences in EEHV occurrence between the different age groups and sexes (p>0.05).

**Table-2:** EEHV test using blood samples in healthy elephants with no record of EEHV (n=84).

Parameter	ELISA/PCR test		Total (n)

	Positive (n)	Negative (n)	
Age			
≤15	36.4% (8/22)	63.6% (14/22)	100% (22)
>15	46.8% (29/62)	53.2% (33/62)	100% (62)
Sex			
Male	55.1% (16/29)	44.8% (13/29)	100% (29)
Female	38.2% (21/55)	61.8% (34/55)	100% (55)

EEHV=Elephant endotheliotropic herpesvirus, PCR=Polymerase chain reaction, ELISA=Enzyme linked immunosorbent assay

## Discussion

In the present study, the occurrence of EEHV in Thai elephants was approximately 40.1%. EEHV can be found in both captive and wild elephants in Thailand. Here, PCR was more sensitive at detecting EEHV in sick elephants, while ELISA was more sensitive at detecting it in carriers.

In the present study, we found EEHV infection (EEHV1 and 4) in three dead wild elephants. To the best of our knowledge, EEHV infection in wild elephants has never been officially reported in Thailand, and its rare occurrence was reported in only a few Asian countries [17,18]. This finding in wild elephants indicates the importance of preventing the transmission of EEHV between captive and wild elephants in Thailand.

In the present study, ELISA-based positivity for EEHV infection was found in 43.9% of healthy elephants. This is consistent with a previous study in Thailand, which used different antigens to survey the EEHV antibody and found an incidence of about 42% [13]. For PCR detection, the present study found infection in 1.2% of blood samples and 5.3% of trunk swabs, which were slightly higher rates than in a previous study that found 0.5% EEHV infection in blood samples [7]. Sample collection in the form of trunk swabs appeared to be more sensitive than that of blood, which is similar to findings in the previous studies that collected samples from trunk wash [8,10,11]. Sampling using trunk swabs is easier than using trunk wash and should be used to routinely monitor EEHV.

The sensitivity of ELISA can be evaluated by detecting the antibody in sera of elephants with a history of EEHV; using this approach, the sensitivity in a previous study was 76% (n=19/25) [12], while that in the present study was 100% (n=7/7). However, the number of PCR-positive cases in this study used to detect the antibody was lower than in the previous study. Moreover, our study collected most samples from Northeast and West of Thailand, where we found 44% ELISA-positive cases, which was higher than in a previous study (37%) in the same area [13].

Although there was no statistically significant difference in the EEHV infection rate in healthy elephants as detected by ELISA between the sexes or age groups, we found that EEHV infection occurred slightly more often in males than in females and in adults rather than in young elephants. This is consistent with the findings of a previous study in Thailand [13]. However, the mortality rate from EEHV was similar between male and female elephants (62-74%) [19].

In our study, EEHV antibody was detected by ELISA in all healthy elephants with previous EEHV infection. This is the first study to combine PCR and ELISA to detect EEHV in Thai elephants. The obtained results are similar to those of a previous study in which a majority (76%) of previously EEHV-infected elephants tested seropositive [12]. However, in the present study, the EEHV IgG antibody could not be detected in all sick elephants, similar to the findings in a previous study [20], suggesting that this could be early or primary infection. We then tracked the antibody of two sick elephants that were PCR positive from the beginning of infection until recovery (PCR negative), but we did not detect the EEHV IgG antibody in them. This suggested that this was the primary infection because, in theory, mammals must generate IgM antibodies, not IgG, to fight EEHV for the 1^st^ time [21]. Nonetheless, we did not further investigate the presence of EEHV IgM due to the lack of available antibodies against elephant IgM; in addition, only one report demonstrating the presence of IgM in elephants has been published [22], and there are no reports regarding elephant IgM-specific disease.

From the records of the Thailand EEHV Taskforce, from 2006 to 2020, 81 elephants were diagnosed with EEHV-HD, among which 56 cases were fatal (69.1%); the majority of dead elephants wereyoung, age less than 15 years (unpublished data). Another study in Thailand using survival analysis suggested that the median age of EEHV presentation is approximately 2 years old, and the mortality rate is as high as 68.97% [19]. We believe that the first EEHV infection in young elephants can be lethal and that young elephants need to generate IgM antibody against this virus. Therefore, the IgG ELISA kit might not be useful in young elephants, indicating the urgent need to develop anti-elephant IgM antibodies and other disease markers such as protein and metabolite markers. For young elephants, behavioral monitoring and PCR analysis must be performed regularly for early detection, to implement early intervention, while the EEHV IgG ELISA kit is useful for screening the occurrence of EEHV in older elephants to identify carriers, to prevent the spread of the disease. In Thailand, we found ELISA/PCR-positive elephants in a herd, which suggests that the virus is already circulating within the herd. In this case, we would encourage the elephants’ owner to routinely perform EEHV monitoring in the blood of young elephants. We suggest performing both PCR and ELISA to increase the sensitivity due to no elephant being positive for both PCR and ELISA at the same time in our study. However, this strategy would increase the cost of elephant management. In Thailand, we currently encourage owners to perform intensive monitoring for the clinical signs of EEHV in young elephants during the at-risk period (younger than 8 years old) instead and also to provide Vitamin C daily to every young elephant to stimulate immune function and reduce mortality.

From the present and previous studies [12,13], many healthy elephants were found to be EEHV seropositive without a previous record of clinical EEHV infection, suggesting that these elephants had survived primary EEHV infection and could be considered as carriers. These carriers have the potential to spread the virus to other uninfected elephants [10]. Therefore, a regular EEHV monitoring strategy is required to prevent the disease from spreading. Alternatively, non-invasively collected samples (e.g., chewed plants and fecal material) could be used to screen for EEHV spreading [23], and retinal imaging has been investigated as a potential rapid screening test for EEHV [24]. Different frequencies of viral shedding in each elephant were previously reported [25], so long-term EEHV monitoring in adult elephants could help us to identify potential viral shedders within a herd. This could be used as information for herd management in the future, such as to separate at-risk calves from potential viral shedders.

## Conclusion

We suggest that PCR is better than ELISA for detecting EEHV in dead or sick elephants and for monitoring EEHV in young elephants, while ELISA is suitable for detecting EEHV carriers, particularly adult elephants. If possible, EEHV screening programs in elephants could use both PCR and ELISA together to increase the sensitivity of the test for elephant herds. The prevalence of EEHV in adult elephants with no record of illness reinforces the need for accurate and timely identification of EEHV carriers within elephant populations to prevent transmission to healthy individuals, especially young elephants with a high risk of mortality from EEHV. The finding of EEHV infection in wild elephants indicates the importance of preventing disease transmission between captive and wild elephants.

## Authors’ Contributions

WW, WJ, and SS collected the samples. PP, BB, WP, and SS performed the detection of EEHV. TW designed the experiment, analyzed the data, and mainly wrote the manuscript. All authors read and approved the final manuscript.
